# A Screening Proposal for Zoom Dysmorphia in Virtual Settings

**DOI:** 10.3390/life13081678

**Published:** 2023-08-02

**Authors:** Cemre Büşra Türk, Fatima N. Mirza, George Kroumpouzos

**Affiliations:** 1 Wellman Center for Photomedicine, Massachusetts General Hospital, Boston, MA 02114, USA; cbusraturk@gmail.com; 2 Department of Dermatology, Harvard Medical School, Boston, MA 02114, USA; 3 Department of Dermatology, Warren Alpert Medical School at Brown University, Providence, RI 02903, USA; fatima_mirza@brown.edu

**Keywords:** zoom dysmorphia, body dysmorphic disorder, screening, scale, questionnaire, telehealth, body image

## Abstract

Zoom dysmorphia (ZD) is a facial dysmorphia that is triggered or aggravated by frequent virtual meetings. The frequent use of videoconferencing platforms has been linked to a distorted perception of facial images as individuals have an increased awareness of their appearance, given constant video feedback. As a result, dysmorphic concerns can develop. It is crucial to identify ZD as this condition interferes with an individual’s life and can trigger or aggravate body dysmorphic disorder (BDD). A standardized approach for screening ZD in non-psychiatric settings has yet to be defined. We discuss the features of ZD and the challenges of screening for ZD in a virtual setting. To facilitate the recognition of ZD in telehealth consultations, we propose a comprehensive ZD screening questionnaire that includes questions related to typical ZD features and a BDD-focused question. The questionnaire is concise and allows the identification of individuals with a potential ZD. A BDD assessment in such individuals should follow.

## 1. Introduction

Zoom dysmorphia (ZD) is a facial dysmorphia that is related to a distorted and distressing self-image triggered or aggravated by frequent video calls and virtual meetings [1,2]. Sufferers of ZD experience anxiety before and during video calls, perceive facial flaws, and believe that others are noticing these flaws. There is concern that ZD can trigger or aggravate body dysmorphic disorder (BDD) [1]. Therefore, identifying ZD in virtual consultations is essential. This article aims to facilitate the provider-patient dialog on dysmorphic concerns (DC) and enhance the recognition of ZD in a telehealth setting. We propose a simple, comprehensive ZD screening questionnaire that practitioners may readily employ.

## 2. ZD Features

Features of ZD largely overlap with those of BDD [3]. BDD is a psychiatric disorder that is classified under obsessive compulsive and related conditions, with an estimated prevalence of 2% in the general population [4]. This disorder consists of a distressing or impaired preoccupation with perceived or real defects, which makes patients fixated on appearance [5]. Focusing excessively on body image impacts people’s functioning negatively in relationships and socializing at work and school [6]. BDD is also associated with high levels of occupational disability, including a loss of productivity. Patients with BDD often present initially to dermatology clinics and request face-to-face or virtual cosmetic consultations for perceived flaws in their appearance. A study identified a five-fold higher prevalence of BDD in patients presenting with skin conditions than controls [7]. Sufferers from BDD often have poor insight and pursue aesthetic procedures to address an imagined/perceived flaw. However, they tend to be dissatisfied with the outcomes, and their concerns intensify after treatment [4]. Diagnosing underlying DC and avoiding inappropriate cosmetic applications is crucial due to potential BDD aggravation and legal consequences for the aesthetic provider. Patients with BDD are more likely to pursue legal action against the provider for malpractice and, in extreme cases, may exhibit physical aggression [8].

Individuals with ZD are concerned about their appearance and scrutinize their facial features, fixating on what they think needs to improve. During virtual meetings, individuals with ZD focus on their on-screen appearance and find facial flaws such as lines, wrinkles, or a big nose. They believe that others notice their perceived flaws. As a result, they view their appearance negatively, which affects their self-esteem and confidence [9]. They experience anxiety about attending video conferences with the camera on. To handle such anxiety, they attempt to look perfect before virtual meetings. Such drastic effects on body dissatisfaction and self-esteem and accompanying anxiety can lead to a desire to seek cosmetic procedures [1].

A recent study utilized a descriptive online questionnaire to explore how university staff members with academic and academic/administrative positions evaluate, perceive, and handle ZD while teaching online [9]. More than half of the participants were concerned about their physical appearance: mainly their faces. This increase in appearance concerns and interest in cosmetic treatments was correlated with increased self-view times. Females were found to have more features of dysmorphia. The study results also showed that sufferers of ZD warranted that their appearances occasionally made them feel insecure and occupationally unstable. Healing mechanisms were pursued to eliminate or, at least, reduce its traits, including hesitation to open the camera, trying to hide or camouflage a perceived flaw with hands, hair, makeup, or clothing, using filter features on the videoconferencing system, and buying a new camera or equipment that helped improve their image. The study concluded that the prevalence of ZD may result in shifting the value away from important attributes (e.g., professionalism, adaptability, collaboration, empathy, and patience) in favor of outside physical appearances.

## 3. Video Conferencing and DC

Virtual meeting platforms (collectively termed “Zoom”) have met a recent surge in popularity [10]. However, such systems can lead to a distorted perception of facial images as individuals have an increased awareness of their appearance, are given constant video feedback, and start focusing on features they perceive as flawed. This may trigger self-criticism and shame. A possible link between people’s perspectives on their facial appearances, increased anxiety, and video conferencing has been described [11].

Rice et al. reported that, despite lockdown measures and decreased in-person appointments, patients sought cosmetic care more frequently during the pandemic because of their appearance in Zoom meetings [1]. Despite being in the middle of the COVID-19 pandemic, over 50% of dermatologists reported a rise in cosmetic consultations in the setting of an increased prevalence of remote work. Notably, 86% of dermatologists surveyed who were fielding these cosmetic concerns reported that their patients referenced video conferencing as the reason for seeking cosmetic consultation. Areas of concern included the forehead/glabellar regions (80%) and periocular areas (78%). More specifically, 77% of providers reported patient cosmetic concerns about upper-face wrinkles, including 64.4% for dark circles under the eyes, 53% for dark facial spots, and 50% for neck sagging. Hart et al. reported that video conferencing was associated with greater concern about appearance and an increased desire for cosmetic surgery and other nonsurgical treatments [12].

Recently, Zoom added a “touch up my appearance” feature, which offers filters to modify one’s appearance during a meeting. This new feature brings Zoom closer to appearance-focused social media (SM [AFSM]) platforms. AFSM platforms offer filters and editing tools to enhance the users’ appearance. Considering that many users are adolescents, some concerns arise, such as feeling pressure to conform to SM-projected beauty standards and developing low self-esteem. The frequent use of AFSM platforms, including Zoom, can be associated with dysmorphic concerns (DC) as the individual focuses on underlying body image issues. Researchers indicated that the frequent use of AFSM platforms is a potential risk factor for BDD [13]. In a study by McLean and colleagues, the time spent on social media was associated with body dissatisfaction and could trigger dysmorphic concerns (DC) in adolescent girls. Activities such as editing images and using filters were also associated with body dissatisfaction [14]. A study by Silence et al. showed that videoconferencing was associated with increased concern about appearance and anxiety upon returning to in-person social activities [15]. The prevalence of anxiety and mental health services was positively correlated with the use of filters in 18- to 24-year-olds.

Sufferers of ZD can be viewed as potential BDD patients. For the diagnosis of BDD, a structured interview followed by a screening scale is mandatory [4,16]. Of note, no validated BDD screening tools have questions related to SM [17], which is a substantial gap. However, screening tools for ZD have yet to be developed, especially as identifying DC in virtual settings can be challenging. Building a trustful and open relationship with patients during virtual visits presents unique challenges. Therefore, developing appropriate screening tools for ZD that include questions that patients can easily understand in virtual consultations is an important first step.

## 4. Challenges in Exploring ZD Virtually

Herein, we comment on the possible challenges to evaluating ZD via telehealth.

- Establishing rapport can be difficult as individuals with ZD may fear being judged or misunderstood. The link between ZD and the interview setting may increase the individuals’ concerns.
- Maintaining eye contact may be difficult due to the person’s fear of being judged or ridiculed because of a perceived flaw in their appearance. It is even more challenging for individuals with ZD in telehealth interviews where eye contact with the physician requires looking at the screen and being exposed to self-images.
- Reticence and silence may occur due to the individual’s avoidant characteristics. This makes evaluating the individual difficult as the conversation is the primary tool for assessment.
- Patients may become easily distracted or disengaged during telehealth visits in the case of technical issues.
- Psychiatric comorbidities can mask DC-related symptoms and cause difficulties in addressing DC remotely and in a short conversation.

## 5. Screening for ZD Virtually

Practitioners should be aware of this condition and the possible challenges of effectively assessing and managing individuals suffering from ZD. The provider’s approach should be tolerant, nonjudgmental, empathetic, and supportive. Multiple telehealth visits may be necessary to establish a rapport, mainly if the patient is reticent, avoidant, or resistant. Establishing a safe and comfortable environment and allowing enough time to discuss patients’ concerns are crucial. Aesthetic providers should make candidates for cosmetic procedures aware that the camera often distorts facial features due to technical aspects (i.e., focal length) [18].

Time may be limited in virtual visits; therefore, using a brief set of questions for screening ZD is essential. Questions tailored to the individual’s behavior in video conferences are extremely helpful in exploring DC virtually.

We propose a three-step screening tool (Table 1). The first step is to ask open-ended questions that facilitate a focused provider–patient dialog. The responses should be evaluated. The second step includes a set of seven ‘yes/no’ questions (Q1 to Q7) that refer to ZD features and the steps taken by the ZD sufferer to reduce those features, such as the use of camouflage to hide a flaw in the appearance or filter of the virtual platform to improve the image [9]. A ‘yes’ response to Q1 (“Are you concerned about facial flaws?”) is required to consider ZD diagnosis. An additional ‘yes’ to one of the remaining questions (Q2 to Q7) indicates possible ZD. In such a case, we proceed with an additional BDD-focused question (included in the structured clinical interview for DSM-5^®^ disorders–clinical version; SCID-5-CV) [19]. Patients who answer ‘yes’ to this question qualify for BDD questionnaires and should be invited to the office for further evaluation. A BDD-focused questionnaire can be sent electronically to those who wish to continue telehealth visits.

In summary, we propose a brief tool that screens ZD in virtual settings. In our experience, individuals can comprehend such questions and respond in a straightforward way, especially as the questions that focus on ZD traits are dichotomous (‘yes/no’). It is crucial to elicit the presence of ZD in the early stage [9]. As ZD is very likely to trigger an increase in BDD, it is essential to identify the presence of BDD in ZD sufferers and treat it appropriately. Our questionnaire can serve as a framework by which the relationship between ZD and BDD can be further studied. This screening tool may prove effective with potential for use in routine practice pending further validation studies.

## Figures and Tables

**Table 1:** ZD screening questionnaire.

**(A) Open-ended questions**
Are you comfortable with being interviewed in a virtual appointment? How do you feel about your appearance during virtual meetings?
(B) Questions for Zoom Dysmorphia screening *
Are you concerned about facial flaws? Do you think that your face is not friendly to the camera? Do you hesitate to open the camera? Have you tried to hide or camouflage your flaw with your hands, hair, makeup, or clothing? Have you sought advice from others to improve your appearance or image? Do you often use the filter features of the video conferencing platform? Did you think about buying a new camera or equipment that helps improve your image?
(C) Body Dysmorphic Disorder-focused question **
In the past month, have you been very concerned that there is something wrong with your physical appearance or the way one or more parts of your body look?

* The questions are adjusted from items in the questionnaire published by Jabali O et al. [9]. ** BDD screening question retrieved from a structured clinical interview for DSM-5^®^ disorders–clinical version (SCID-5-CV) by the American Psychiatric Association [19].

## Data Availability

The data of this study are contained within the article.
